# Synergistic Effects of Multi-Kinase Inhibition on *LRRK2*-G2019S and Alpha-Synuclein Pathologies in Models of Parkinson’s Disease

**DOI:** 10.3390/biomedicines14040927

**Published:** 2026-04-18

**Authors:** Xiaoguang Liu, Sean Baxley, Michaeline L. Hebron, Charbel Moussa

**Affiliations:** Translational Neurotherapeutics Program, Laboratory for Dementia and Parkinsonism, Department of Neurology, Georgetown University Medical Center, Washington, DC 20057, USA; xl371@georgetown.edu (X.L.); sdb82@georgetown.edu (S.B.); mlh88@georgetown.edu (M.L.H.)

**Keywords:** Parkinson’s disease, alpha-synuclein, LRRK2, dopamine, tyrosine kinase inhibition

## Abstract

**Introduction**: Pathogenic mutations in leucine-rich repeat protein kinase-2 (*LRRK2*), particularly G2019S, constitute the most common cause of autosomal dominant PD. **Methods**: Mouse models encoding human mutant alpha-synuclein (*SNCA A53T*) and *LRRK2* G2019S were treated with a brain-penetrant kinase inhibitor (BK40196). Behavior, nigrostriatal and mesolimbic dopamine (DA) pathways were examined. **Results**: Mice harboring *LRRK2 G2019S* do not show age-dependent motor symptoms, but mice encoding *SNCA* A53T display motor deficits, while both strains exhibit anxiety-like behavior and BK40196 improves motor and behavioral defects. BK40196, a multi-kinase inhibitor of Abelson (Abl), Discoidin domain receptor (DDR)-1, c-KIT and FYN, alters microglial morphology and alpha-synuclein levels in *SNCA* A53T mice and improves DA neurotransmission, primarily via the nigrostriatal system. BK40196 inhibits brain LRRK2 G2019S (IC_50_ of 89nM) and does not affect phosphorylated or total peripheral LRRK2 levels (lungs, kidneys, liver, etc.). *LRRK2* G2019S mice treated with BK40196 exhibit distinct increases in DA in mesolimbic neurons such as the nucleus accumbens (NAcc), suggesting differential mechanisms of DA neurotransmission in mutant alpha-synuclein and *LRRK2* models of PD. **Conclusions**: *LRRK2* G2019S may primarily involve mesolimbic pathways leading to nonmotor symptoms independent of the motor and behavioral manifestations associated with alpha-synuclein via the nigrostriatal system. BK40196 may provide a comprehensive and synergistic therapeutic approach that addresses multiple mechanisms to reduce the pathologies related to *LRRK2* G2019S and/or *SNCA* in PD. The multiple pathologies of PD necessitate a holistic approach that simultaneously targets inflammation and autophagy and LRRK2 inhibition.

## 1. Introduction

Parkinson’s disease (PD) is a progressive disorder primarily characterized by loss of dopaminergic neurons in the substantia nigra *pars compacta* (SN), leading to motor deficits such as bradykinesia, rigidity, and resting tremors. In addition to motor symptoms, PD is associated with nonmotor complications, including cognitive decline, anxiety, and depression [1]. Despite extensive research, the exact underlying molecular mechanisms driving PD remain incompletely understood, and disease-modifying therapies are lacking. The defining pathological feature of PD is intracellular aggregation of α-synuclein in Lewy bodies and Lewy neurites. α-synuclein, a presynaptic protein involved in synaptic plasticity and vesicle trafficking, undergoes misfolding and aggregation, leading to neuronal dysfunction, synaptic loss, and ultimately cell death [2]. Mutations in genes such as *SNCA* (e.g., *SNCA* A53T), which encodes α-synuclein, account for most cases of sporadic PD. *lrrk2,* which encodes leucine-rich repeat protein kinase-2 (e.g., *LRRK2* G2019S), is the most common genetic mutation of PD [3,4]. The underlying mechanisms of *LRRK2*-PD range from inflammation and autophagy to some Alzheimer’s disease (AD) pathologies, like tau hyperphosphorylation [5,6,7], impairment of vesicular trafficking and autophagic degradation and defective α-synuclein clearance [8,9,10]. This suggests that *LRRK2* mutations contribute to PD via distinct but overlapping mechanisms with α-synuclein, particularly through disruption of the autophagy–lysosome system [11,12].

Some *LRRK2*-mutant PD cases lack α-synuclein pathology, raising questions about whether these mutations drive neurodegeneration independently or synergistically with α-synuclein. We recently reported BK40196, a novel multi-kinase inhibitor [13] that potently and selectively targets tyrosine kinases like Abl, FYN, DDR1 and c-KIT, which display synergistic effects on pathological features, like autophagy, inflammation and DA metabolism, as potential therapeutic strategies for PD. Importantly, feasibility clinical studies have shown that nilotinib (DDR1 inhibitor) and bosutinib (Abl-Src inhibitor) not only enhance DA metabolism but also significantly reduce phosphorylated tau in AD, PD, and Lewy Body Dementia (LBD) patients, coinciding with behavioral and functional improvement [14,15]. The current study investigates the effects of the multi-kinase inhibitor BK40196 in PD models carrying *LRRK2* G2019S or *SNCA* A53T mutations. Because *LRRK2* G2019S mice do not express alpha-synuclein and do not exhibit dopaminergic cell death or inflammation, we used aged *SNCA* A53T mice to study the effects of BK40196 in a model of alpha-synucleinopathy. We examined whether BK40196 modulates LRRK2 signaling, dopaminergic neurotransmission, neuroinflammation, and behavioral phenotypes in mesolimbic and nigrostriatal pathways.

## 2. Materials and Methods

### 2.1. Kinase Screening and IC_50_ and Kd Evaluation

The KINOMEscan™ screening platform (Eurofins DiscoverX Corporation, San Diego, CA, USA) was utilized to quantitatively measure interactions between BK40196 and BK40195 and more than 450 human kinases, as we previously reported [16]. Individual IC_50_ and Kd values were determined against each of the selected kinases identified in KINOMEscan™, including DDR1, c-KIT mutant, Abl, FYN, wild-type LRRK2 and LRRK2 G2019S, using Eurofins’ standard KinaseProfiler, IC_50_ Profiler™ and Kd Profiler™ assays (Eurofins Cerep, Paris, France). Radioligand binding and functional assays (*n* = 3) were then used to evaluate the activity of BK40196 using reference standards of each assay to ensure the validity of the results obtained against a nonspecific vesicular monoamine transporter (VMAT), DA transporter (DAT), DA receptor D1, serotonin transporter (SERT) and norepinephrine transporter (NET) (Eurofins Advinus Discovery, Bangalore, India).

### 2.2. Transgenic Mice and Treatment

Male and female *LRRK2* G2019S knock-in mice, aged (8–10 months, *n* = 7–8 per group) and young (3–4 months, *n* = 7–8 per group), were treated with BK40196 (20 mg/kg, i.p., daily for 4 weeks) or DMSO vehicles, and phosphorylation of LRRK2 at Ser1292 was measured. Male and female (8–12 months, *n* = 8 per group) *SNCA* A53T mice were treated with LRRK2 inhibitor BK40196 (20 mg/kg, i.p. daily for 4 weeks) or BK40195, as a non-inhibitor of LRRK2 (25 mg/kg, i.p. daily for 4 weeks), or DMSO vehicles. Behavioral tests (*n* = 7–8) were performed for 4 weeks post-drug administration. Upon conclusion of behavioral studies, all animals were sacrificed, and tissues were collected and analyzed via immunohistology and/or WB (*n* = 5–7).

All analyses were performed as within-genotype comparisons between vehicle- and drug-treated animals. Wild-type littermates were not included in all experiments, as the primary goal was to assess pharmacological modulation within established disease-relevant genotypes rather than baseline disease causation.

### 2.3. Rotarod Test

Mice (*n* = 7–8) were placed on an accelerating rotarod (Columbus Instruments, Columbus, OH, USA) and trained to stay at a constant 5 rpm rotation up to 5 min; then, the speed of the rotarod was incrementally increased by 0.2 rpm/min and the latency to fall off the rod was measured using an individual timer for each mouse. Mice were trained for 3 consecutive days before the final measurement.

### 2.4. Elevated Plus Maze Test

An elevated, plus-shaped (+) apparatus measures anxiety in general with two open and two closed arms, assessing the general aversion of mice to open spaces. Anxiety was assessed by individually placing mice (*n* = 7–8) in the center of the apparatus, and a blinded observer counted the entries of mice into open arms and/or closed arms, as well as time spent in each and the total distance traveled over a 10 min period.

### 2.5. Immunohistochemistry

Animals were deeply anesthetized with a mixture of Xylazine and Ketamine (1:8), washed with normal saline for 1 min and then perfused with 4% paraformaldehyde (PFA) for 15 min. Brains were isolated and quickly placed in 4% PFA for 24 h at 4 °C and then stored in 30% sucrose at 4 °C for 48 h. Brain tissues were cut using a cryostat microtome into 20 µm thick coronal sections and stored at −20 °C. Microglia were stained using rabbit anti-Iba1 antibody (Cat No: 019-19741, Fuji, Japan). VMAT1 was stained using mouse anti-VMAT1 antibody (Cat No: sc-166391, Santa Crutz, Dallas, TX, USA). VMAT2 was stained using rabbit anti-VMAT2 antibody (Cat No: PA5-22864, Invitrogen, Carlsbad, CA, USA). DAT was stained using mouse anti-DAT1 antibody (Cat No: NBP2-22164, Novus Biologicals, Centennial, CO, USA). D1 was stained using rabbit anti-D1 antibody (Cat No: GTX100354. GeneTex, Irvine, CA, USA). Anti-tyrosine hydroxylase (TH) was stained using rabbit polyclonal antibody (Cat No: AB152, Sigma-Aldrich, St Louis, MO, USA). Nuclear staining with 4′,6-diamidino-2-phenylindole (DAPI) was performed according to the manufacturer’s protocol (Life Technologies, Miami, FL, USA).

### 2.6. Western Blot

Tissues were collected one hour after the final dose. Animals were deeply anesthetized and transcranially perfused with saline prior to tissue collection. Brain regions were rapidly dissected; tissues designated for biochemical analyses were snap-frozen, while those for histological analysis were post-fixed as described above. Soluble proteins from the brain, liver, lungs and kidneys were extracted from mouse lysates via homogenization in 1x STEN buffer (50 mM Tris (pH 7.6), 150 mM NaCl, 2 mM EDTA, 0.2% NP-40, 0.2% BSA, 20 mM PMSF and protease cocktail inhibitor), centrifuged at 10,000× *g* for 20 min at 4 °C. Protein quantification assays were performed and protein levels were normalized in the homogenized tissue samples prior to Western blot (WB) and ELISA assays. An equal amount of protein for the assays was quantified using standard graphs. Extracts were analyzed by WB on 4–12% Criterion™ XT Bis-Tris Protein Gel (Cat#3450125, Bio-Rad, Philadelphia, PA, USA). Beta-actin (β-actin) was probed (1:3000) with monoclonal antibody (Cat# MAB1501R, Sigma-Aldrich, St Louis, MO, USA). Human alpha-synuclein and oligomeric synuclein were probed (1:4000) with monoclonal antibody (Thermo Fisher, AHB0261, Rockford, IL, USA). Phospho-c-KIT was probed (1:1000) with polyclonal antibody (Cat No: 44-496G Invitrogen, Invitrogen, Carlsbad, CA, USA). Phosphorylated LRRK2 (phospho S1292) was probed with rabbit monoclonal antibody (Cat# ab203181, Abcam, Cambridge, UK) and total LRRK2 was probed with rabbit monoclonal antibody (Cat# ab133474, Abcam, Abcam, Cambridge, UK). WBs were quantified by densitometry using Quantity One 4.6.3 software (Bio-Rad, Hercules, CA, USA) and Image J (Vesrion 1.54).

### 2.7. ELISA

Soluble brain, kidney, lung and liver protein lysates were used. A specific total mouse LRRK2 ELISA kit was used (Cat# A312264, Antibodies.com, St Louis, MO, USA). A dopamine ELISA kit (Cat# MBS269234, MyBiosource, San Diego, CA, USA) and HVA ELISA kit (Cat # MBS1601074, MyBiosource, San Diego, CA, USA) were used. ELISAs were performed according to the manufacturer’s protocol on soluble tissue extracts from brain, kidney, lung and liver lysates in 1XSTEN buffer.

### 2.8. Microglial Morphological Analysis

In this analysis, 63x z-stack images (0.19 μm intervals) of IBA1-stained microglia from treated and untreated mice were collected on a Zeiss LSM confocal microscope. 3D images were then analyzed using the “surfaces” plug-in in Imaris 10.0 to ascertain cell surface area. Individual microglial surface areas were calculated in the advanced statistics tab of Imaris.

### 2.9. Silver Staining

Silver staining was performed using an FD NeuroSilver Kit II (Cat No: PK 301A, FD NeuroTechnologies, INC, Columbia, MD, USA) according to the manufacturer’s protocols.

### 2.10. Statistical Analysis

All statistical analyses were performed using GraphPad Prism version 10 (GraphPad software, Inc., San Diego, CA, USA). One-way analysis of variance (ANOVA) (and nonparametric or mixed) tests were used for comparison of means of multiple groups. A two-tailed Student’s *t*-test (and nonparametric tests) was used for comparison of means of two groups. The Shapiro–Wilk test was used for checking normality before performing statistic tests. Unless otherwise indicated, data are expressed as the mean ± SD. Statistical tests and significance levels are indicated with an exact *p*-value for every comparison that either shows a significant difference and/or a strong trend.

## 3. Results

### 3.1. BK40196 Inhibits LRRK2 G2019S

**Figure 1:**
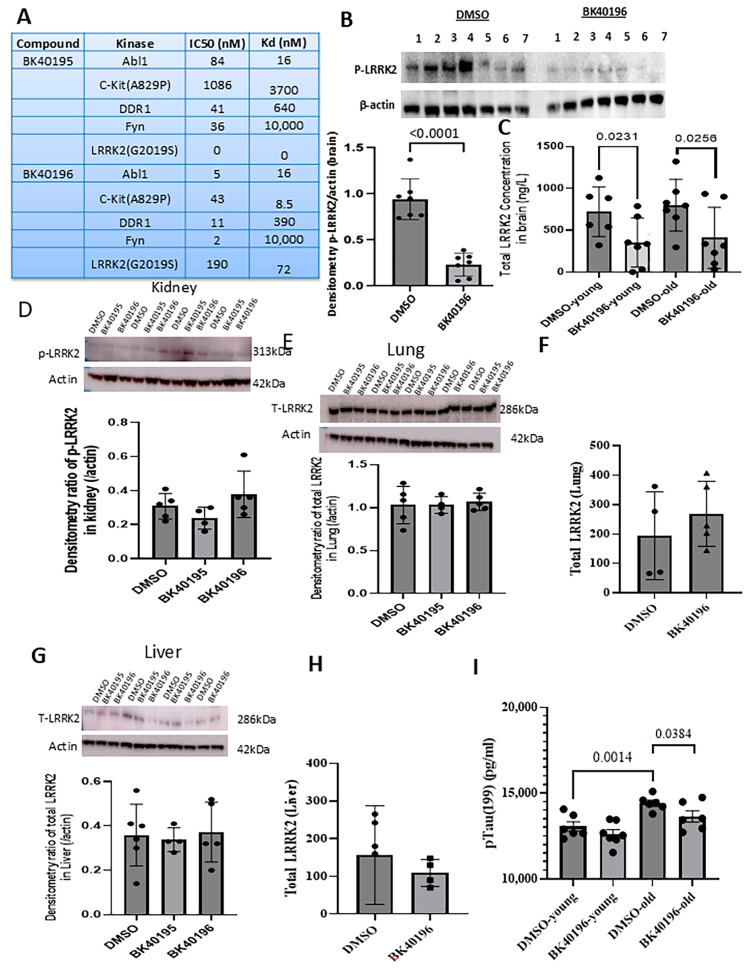
BK40196 selectively inhibits central but not peripheral LRRK2 in LRRK2-G2019S mice. (**A**) In vitro kinome kinase inhibition profiles of BK40195 and BK40196. (**B**) Western blot analysis of brain lysates from young and old *LRRK2* G2019S mice treated with DMSO or BK40196, showing a significant reduction in p-LRRK2 in old *LRRK2* G2019S mice following BK40196 treatment. (**C**) ELISA demonstrates marked decreases in total LRRK2 in both young and old *LRRK2* G2019S mice after BK40196 treatment. (**D**–**H**) Western blot and ELISA analyses of kidney, liver, and lung samples show no detectable effects of BK40196 on phosphorylated LRRK2 in kidney or on total LRRK2 in lung and liver. Phosphorylated LRRK2 was not detected in lung or liver tissues. (**I**) ELISA demonstrates a marked increase in total hyperphosphorylated tau levels in old *LRRK2* G2019S mice, and this effect was reversed after BK40196 treatment. All analyses represent within-genotype comparisons between vehicle- and drug-treated mice. Statistical analysis used two-tailed Student’s *t*-tests. *N* = 5–7 animals per group.

We previously reported the synthesis of BK40195 and BK40196 and performed a pharmacokinetic (PK) analysis showing that BK40196 reached peak concentration in the serum at 1 h and in the brain at 2 h following I.P. injection with a serum: brain ratio greater than 30%, at 20 mg/kg, indicating that this molecule readily enters the CNS [13]. Additionally, previous kinome screening studies indicated that a few kinases are commonly targeted by both BK40195 and BK40196, including Abl, c-Kit (including mutants), DDR1 and FYN (Figure 1A); while BK40196 inhibits *LRRK2* G2019S mutants at IC_50_ = 190 nM and Kd = 72 nM, BK40195 does not target *LRRK2* or its mutation. To further validate the in vivo efficacy of BK40196 as an LRRK2 inhibitor versus BK40195 as a non-inhibitor of LRRK2, we treated 8–10-month-old G2019S knock-in mice with BK40196 (20 mg/kg, i.p., daily for 4 weeks) and assessed phosphorylation of LRRK2 at Ser1292. BK40196 treatment resulted in a significant reduction in pSer1292-LRRK2 (Figure 1B) in the brain. Further analysis with ELISA confirmed that while no changes in total LRRK2 were detected between young (2–3 months) and old (8–10 months) mice (Figure 1C), BK40196 significantly reduced total levels of LRRK2, which include phosphorylated and non-phosphorylated LRRK2. To evaluate the potential effects of BK40196 versus BK40195 on inhibition of LRRK2 in peripheral organs, including kidneys, we performed WB analysis and observed no effects on phosphorylated LRRK2 (Figure 1D). Phosphorylated LRRK2 was not detected in lungs, and BK40196 had no effects on total LRRK2 via WB (Figure 1E) or ELISA (Figure 1F). Similarly, phosphorylated LRRK2 was not detected in the liver, and BK40196 had no effects on total LRRK2 (Figure 1G,H). Interestingly, tau hyperphosphorylation was significantly increased in old vs. young *LRRK2* G2019S mice (Figure 1I), and BK40196 significantly reduced tau phosphorylation, consistent with previous data showing profound BK40196 effects on tau pathology [13].

### 3.2. BK40196 Reduces Alpha-Synuclein Levels and Counteracts Its Pathology

**Figure 2:**
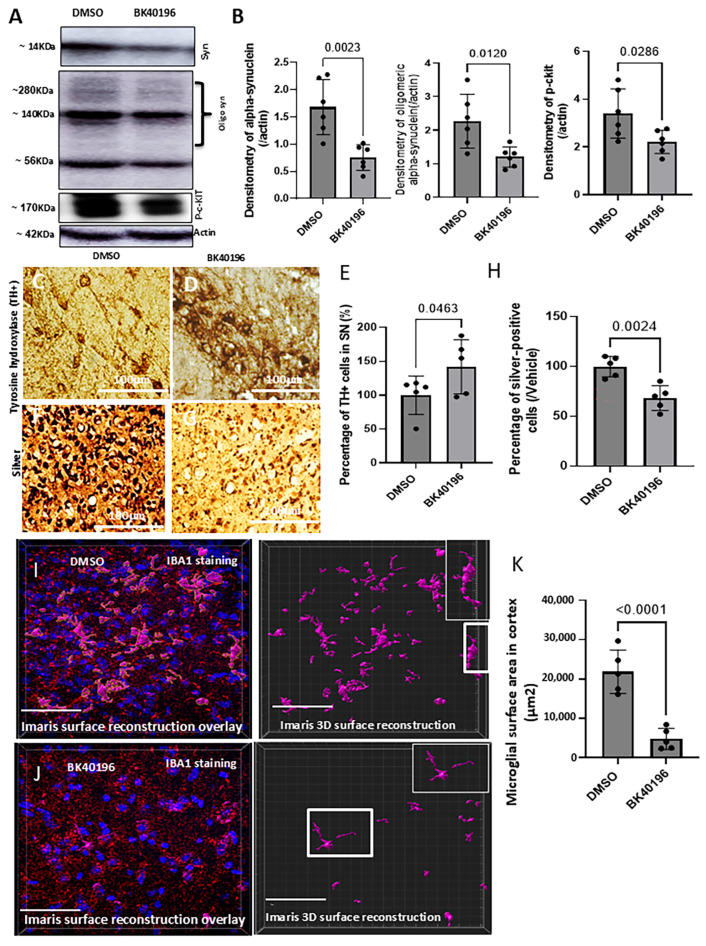
BK40196 reduces α-synuclein levels and mitigates α-synuclein-related pathology. *SNCA* A53T mice (7.6–12.5 months) were treated with vehicle or BK40196 (20 mg/kg/day, i.p.) for 4 weeks. (**A**,**B**) Western blot analysis of brain lysates shows reductions in α-synuclein, oligomeric α-synuclein, and p-c-KIT, with corresponding densitometric quantification. (**C**–**E**) Immunohistochemistry of substantia nigra sections demonstrates TH-positive neurons co-labeled with Nissl staining, with optical density quantification. (**F**–**H**) Silver staining of the cortex shows reduced silver-positive cells after BK40196 treatment, with corresponding cell counts. (**I**–**K**) IMARIS 3D reconstruction of Iba1-positive microglia reveals a larger surface area and amoeboid morphology in DMSO-treated mice, whereas BK40196-treated mice display more ramified microglia, confirmed by quantitative surface-area measurements. Data are expressed as the mean ± SD. Statistical analysis used two-tailed Student’s *t*-tests. *N* = 5–6 animals per group.

Because *LRRK2* G2019S mice do not express alpha-synuclein and do not exhibit dopaminergic cell death or inflammation, we used aged *SNCA* A53T mice (8–12 months old) to study the effects of BK40196 in another model of PD that is reminiscent of alpha-synucleinopathy. BK40196 significantly reduced the levels of monomeric as well as oligomeric alpha-synuclein and reduced the level of phosphorylated (activated) c-Kit (Figure 2A,B), consistent with our previous data that BK40196 inhibits c-Kit, as well as Abl and Fyn [13]. BK40196 improved the staining level of tyrosine hydroxylase-positive (TH^+^) neurons in the SN (Figure 2D,E) and reduced cell death via silver staining (Figure 2G,H) compared to DMSO (Figure 2C,F). Microglial activity was evaluated using confocal microscopy to assess IBA-1-stained microglial shapes and surface areas in DMSO-treated *SNCA* A53T mice (Figure 2I, first panel, 12–14 months), which showed significantly larger microglial surfaces (Figure 2I,K, second panel) with bigger cell bodies (Figure 2I, insert) compared to BK40196-treated mice (Figure 2J,K).

### 3.3. BK40196 Improves Motor Performance and Anxiety-like Behavior in Mice

**Figure 3:**
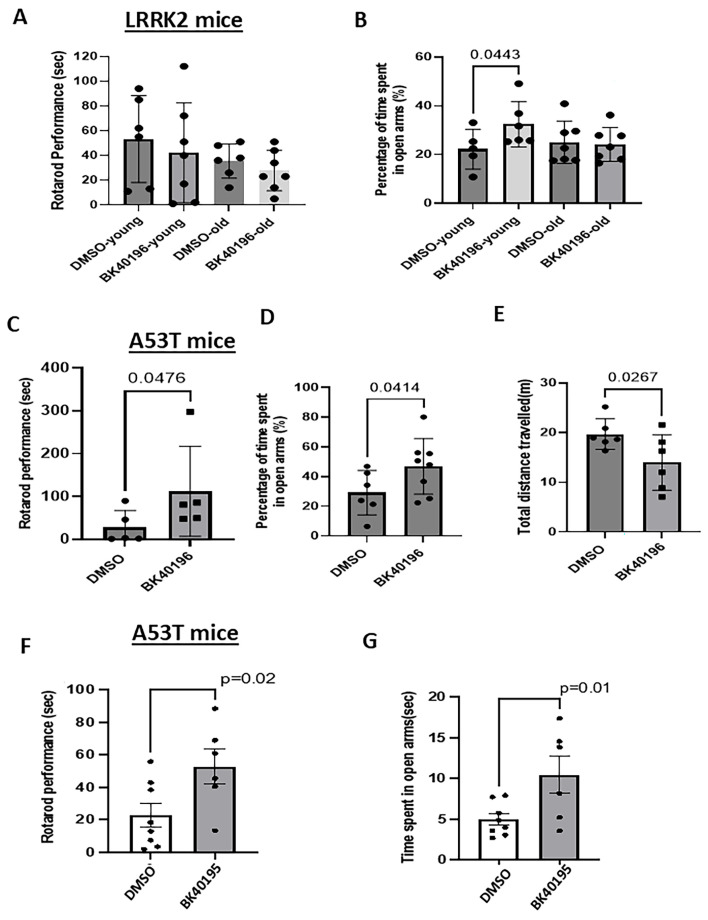
BK40196 improves motor performance and reduces anxiety-like behaviors in mouse models. (**A**) Rotarod performance shows *LRRK2* G2019S mice show no age-dependent changes in rotarod performance. (**B**) In the elevated plus maze, BK40196 (daily 20 mg/kg, i.p. for 4 weeks) reduces anxiety-like behavior by increasing the percentage of time spent in open arms in young *LRRK2* G2019S mice, but not in old *LRRK2* G2019S mice. (**C**) Rotarod shows improved motor performance in *SNCA* A53T mice treated with BK40196 (daily 20 mg/kg, i.p. for 4 weeks) compared with vehicle. BK40196 (**D**) reduces anxiety-like behavior by increasing the percentage of time spent in open arms and (**E**) significantly reduces total distance travelled in the elevated plus maze in *SNCA* A53T mice. (**F**,**G**) In *SNCA* A53T mice, BK40195 improves rotarod performance and increases open-arm time. *N* = 5–7 animals per group.

We compared the effects of BK40196 on behavioral symptoms in *LRRK2* G2019S versus *SNCA* A53T mice. *LRRK2* G2019S mice did not display any changes in motor performance as they aged (Figure 3A) or any changes in anxiety-like behavior (Figure 3B). However, BK40196 increased the time young mice spent in the open arms (Figure 3B), suggesting reduced anxiety. BK40196 significantly improved motor performance in *SNCA* A53T mice (Figure 3C) and increased time spent in open arms (Figure 3D). It was also noticeable that BK40196 increased the distance travelled by *SNCA* A53T mice (Figure 3E). In comparison to BK40196, BK40195 improved motor performance (Figure 3F) and reduced anxiety (Figure 3G) in *SNCA* A53T mice.

### 3.4. BK40196 Increased Dopamine Levels in LRRK2-G2019S and SNCA A53T Mice

**Figure 4:**
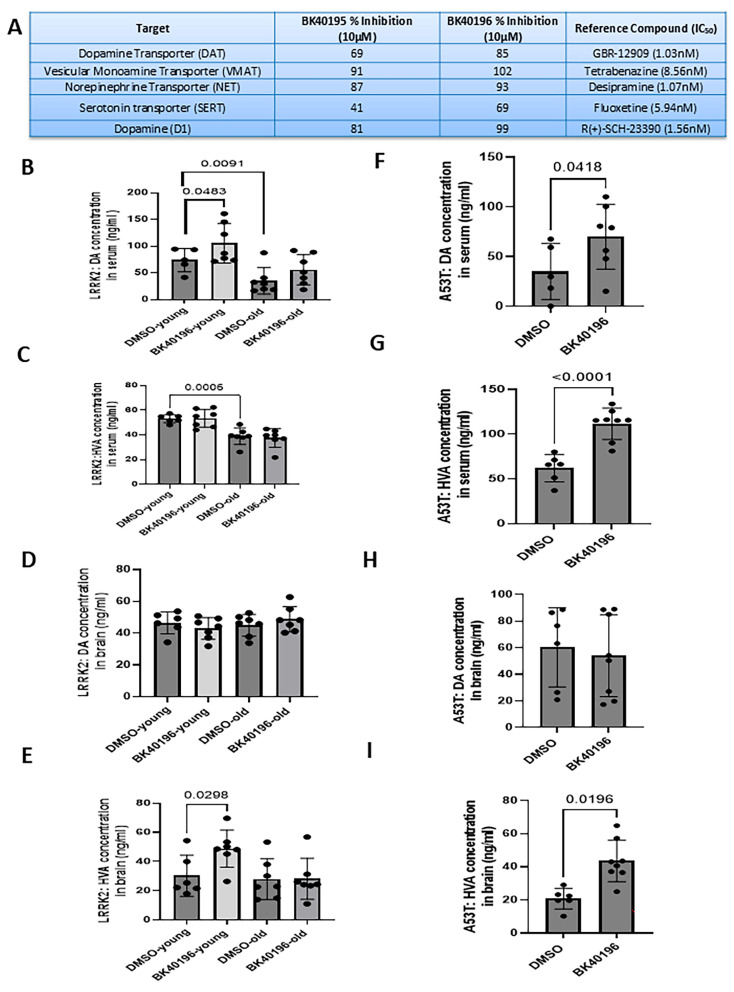
BK40196 increases dopamine levels in LRRK2-G2019S and SNCA A53T mice. (**A**) Radioligand inhibition assays showing BK40195 and BK40196 inhibition of VMAT vesicular transporters, DAT, NET, SERT, and D1 receptors in vitro. (**B**–**E**) ELISA quantification of serum and whole-brain dopamine (DA) and homovanillic acid (HVA) in *LRRK2* G2019S mice shows age-related reductions in DA/HVA and increases following BK40196 treatment, particularly in serum. (**F**–**I**) Serum and brain DA/HVA levels in *SNCA* A53T mice demonstrate increased values after BK40196 treatment. Statistical analysis used two-tailed Student’s *t*-tests. Data are presented as the mean ± SD; *p* < 0.05 unless otherwise indicated. *N* = 6–7 animals per group.

To better understand the effects of *LRRK2* versus alpha-synuclein mutations on dopaminergic transmission, we thoroughly investigated the status of DA metabolism in corresponding mice and examined the effects of BK40196. Radioligand studies in vitro (Figure 4A) showed that both BK40195 and BK40196 significantly inhibited nonspecific VMAT vesicular transporters and DA and D1 receptors as well as monoamine transporters DAT, NET and SERT. In vivo studies showed that the levels of DA (Figure 1B) and its metabolite HVA (Figure 4C) were diminished in the serum of *LRRK2* G2019S mice as they age (Figure 4B), and BK40196 increased the level of DA in the serum of both young and old mice (Figure 4B). No changes in DA levels were detected in the brains of *LRRK2* G2019S mice (Figure 4D), but BK40196 increased the level of HVA in young mice (Figure 4E). Taken together, these studies may indicate a reduction in DA levels in the presence of LRRK2 mutation, but BK40196 increases DA levels. BK40196 increased the serum levels of both DA (Figure 4F) and HVA (Figure 4G) in SNCA A53T mice. No changes were detected in DA levels in the brain (Figure 4H), but HVA was significantly increased (Figure 4I).

### 3.5. BK40196 Differentially Increases D1 Expression in LRRK2 G2019S Mice

**Figure 5:**
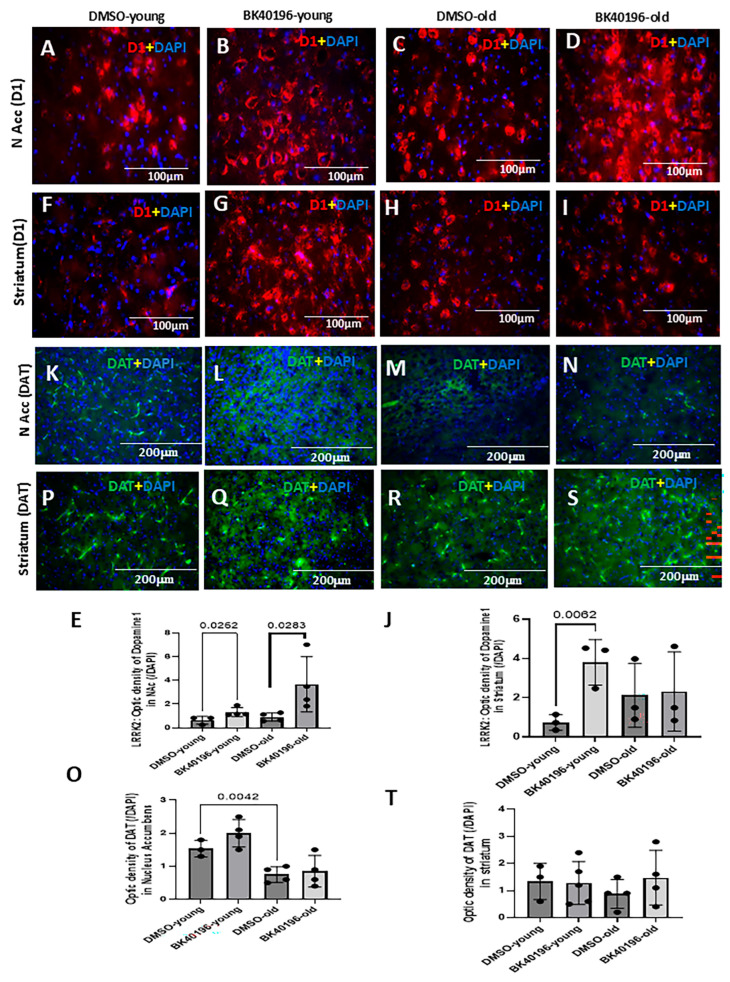
BK40196 differentially enhances D1 receptor expression in *LRRK2*-G2019S mice. (**A**–**D**) Representative D1 receptor (D1 + DAPI) immunostaining in the nucleus accumbens (NAcc) of young and old *LRRK2* G2019S mice shows increased D1 signal after BK40196 treatment. (**E**) Quantification of NAcc D1 optical density. (**F**–**I**) D1 receptor staining in the striatum demonstrates increased expression in young but not old *LRRK2* G2019S mice after BK40196 treatment. (**J**) Quantification of striatal D1 optical density. (**K**–**T**) DAT immunostaining demonstrates age-related decreases in the NAcc but no age-related changes in the striatum. BK40196 does not alter DAT expression. Data represent the mean ± SD. Statistical analysis used two-tailed Student’s *t*-tests. *N* = 3–4 per group.

We then dissected the effects of BK40196 on the mesocortical compared to nigrostriatal systems in *LRRK2* G2019S versus *SNCA* A53T mice. Staining of DA D1 receptors in the nucleus accumbens (NAcc), a major dopaminergic nucleus of the mesolimbic system, showed no difference between young (Figure 5A,E) and old (Figure 5C,E) *LRRK2* G2019S mice. However, a significant increase in D1 expression was observed in young (Figure 5B,E) and old (Figure 5D,E) mice treated with BK40196. Staining D1 receptors in the striatum also showed no difference between young (Figure 5F,J) and old (Figure 5H,J) *LRRK2* G2019S mice. However, a significant increase in BK40196-treated mice was observed only in young (Figure 5G,I), but not old, mice (Figure 5I,J). DAT was significantly reduced in old (Figure 5M,O) versus young (Figure 5K,O) *LRRK2* G2019S mice in the NAcc, while DAT expression was unchanged between young (Figure 5P,T) and old (Figure 5R,T) mice in the striatum, suggesting loss of DA transmission in the mesolimbic pathway. BK40196 did not alter the level of DAT expression (Figure 5K–T) in old (Figure 5R,T) mice in the striatum, suggesting loss of DA transmission in the mesolimbic pathway. BK40196 did not alter the level of DAT expression (Figure 5K–T).

### 3.6. BK40196 Differentially Alters VMAT Expression in LRRK2 G2019S

**Figure 6:**
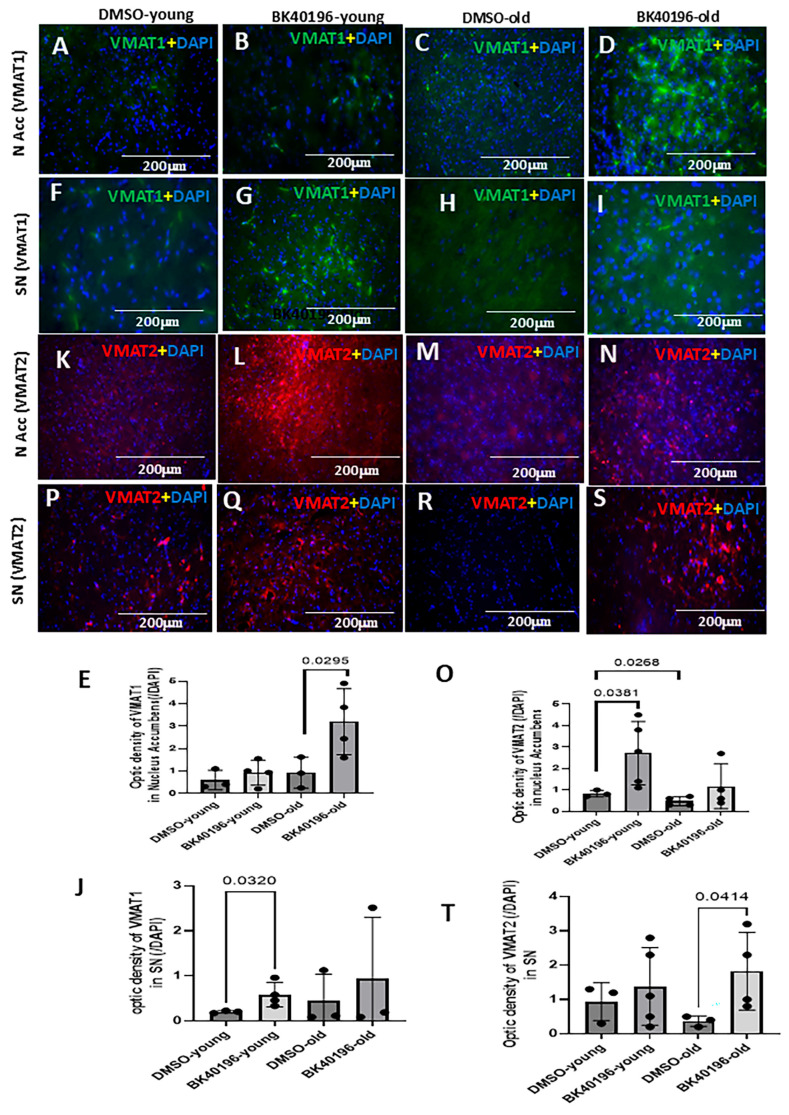
BK40196 differentially modulates VMAT1 and VMAT2 expression in *LRRK2*-G2019S mice. (**A**–**E**) VMAT1 immunostaining in the NAcc shows no age-related differences but increased VMAT1 expression in old mice following BK40196 treatment. (**F**–**J**) VMAT1 staining in the substantia nigra (SN) shows increased expression in young mice following BK40196 treatment. (**K**–**O**) VMAT2 staining in the NAcc shows reduced VMAT2 in young versus old mice; BK40196 restores VMAT2 levels in young mice only. (**P**–**T**) VMAT2 staining in the SN shows increased expression in old mice after BK40196 treatment. Optical density values are shown as the mean ± SD. Statistical analysis used two-tailed Student’s *t*-tests. *N* = 3–4 per group.

The expression level of VMAT1 was unchanged between young (Figure 6A,E) and old (Figure 6C,E) in the NAcc of *LRRK2* G2019S mice, and BK40196 increased VMAT1 expression in old (Figure 6D,E) but not young mice (Figure 6B,E). The expression level of VMAT1 was also unchanged between young (Figure 6F,J) and old (Figure 6H,J) in the SN of *LRRK2* G2019S mice, and BK40196 increased VMAT1 expression in young (Figure 6G,J) but not old mice (Figure 6I,J). The expression level of VMAT2 was significantly reduced in young (Figure 6K,O) compared to old mice (Figure 6M,O) in the NAcc of *LRRK2* G2019S mice, and BK40196 increased VMAT2 expression in young (Figure 6L,O) but not old mice (Figure 6N,O). The expression level of VMAT2 was also unchanged between young (Figure 6P,T) and old (Figure 6R,T) in the SN of *LRRK2* G2019S mice, and BK40196 increased VMAT2 expression in old (Figure 6S,T) but not young mice (Figure 6Q,T).

### 3.7. BK40196 Differentially Modulates Dopaminergic Markers Across Nigrostriatal and Mesolimbic Circuits in SNCA A53T Mice

To further dissect the circuit-specific effects of BK40196 in α-synuclein-driven pathology, we performed additional immunohistology analyses of DA receptor and transporter (DAT) expression in the striatum, NAcc, and SN of *SNCA* A53T mice treated with BK40196 at a daily dose of 20 mg/kg i.p. for 4 weeks. In the striatum, BK40196 treatment resulted in a significant increase in D1 receptor expression compared to vehicle-treated *SNCA* A53T mice (Supplementary Figure S1A,B,G, *n* = 4–5 per group). In contrast, no changes in D1 receptor levels were observed in either the NAcc or the SN (Supplementary Figure S1H,I) following BK40196 treatment. Analysis of presynaptic dopaminergic markers revealed that BK40196 significantly increased DAT (Supplementary Figure S1C,D,L) and vesicular monoamine transporter 2 (VMAT2) (Supplementary Figure S1E,F,O) expression in the SN of *SNCA* A53T mice. No significant changes in DAT or VMAT2 expression were detected in the striatum or NAcc (Supplementary Figure S1J,K,M,N). Expression of VMAT1 remained unchanged across all three regions examined (Supplementary Figure S1P–R).

## 4. Discussion

The underlying mechanisms of *LRRK2*-PD range from inflammation and autophagy to some AD pathologies, notably tau phosphorylation and accumulation [5]. The clinical phenotype of *LRRK2*-PD—like typical PD—involves both motor and nonmotor symptoms and levodopa response. Our results primarily indicate that BK40196 targets motor and nonmotor symptoms in PD models and synergistically inhibits *LRRK2* G2019S as well as other pathophysiology, including alteration of microglial morphology, suggesting reduction of the microglial activation state in *SNCA* A53T mice and tau de-phosphorylation in *LRRK2* G2019S, consistent with previous findings in rTg4510 mice [13]. Recent research suggests that *LRRK2* G2019S and *SNCA* A53T affect distinct neural pathways, potentially explaining variability in PD symptomatology. The nigrostriatal pathway, which regulates voluntary movement, is particularly vulnerable to alpha-synuclein-driven toxicity, leading to classical PD motor symptoms [17,18]. Studies using *LRRK2* G2019S transgenic or knock-in mouse models have demonstrated anxiety- and depression-like behaviors, notably prior to the onset of motor impairments [19,20,21]. Moreover, stress-induced anxiety phenotypes are accompanied by changes in c-Fos activation within the medial prefrontal cortex and NAcc, which are key nodes of the mesolimbic pathway [5]. This distinction is supported by our findings that *LRRK2* G2019S mice do not exhibit motor deficits, while *SNCA* A53T mice show motor impairments.

This study reveals a novel insight into understanding PD heterogeneity by attempting to dissect the distinct and overlapping contributions of *LRRK2* (G2019S) and α-synuclein in mesolimbic and nigrostriatal pathways. The current data show that *LRRK2* G2019S exhibited prominent alterations within the mesolimbic pathway, including changes in D1, DAT, and VMAT2 expression in the NAcc but minimal or absent alterations in the striatum and SN (Supplementary Table S1). BK40196 preferentially increased DA markers in mesolimbic regions of *LRRK2* G2019S, consistent with behavioral data showing the presence of anxiety-like and nonmotor phenotypes in the absence of overt motor deficits.

In contrast, *SNCA* A53T pathology preferentially disrupts the nigrostriatal pathway, consistent with classical PD motor dysfunction. BK40196 restores dopaminergic function in this circuit by increasing DAT and VMAT2 expression in the SN, suggesting improved DA synthesis, vesicular packaging, and presynaptic neuronal integrity, and upregulation of D1 receptor expression in the striatum, indicating enhanced postsynaptic responsiveness to DA release. Notably, BK40196 had no effect on D1, DAT, VMAT2, or VMAT1 expression in the NAcc of *SNCA* A53T, indicating that mesolimbic dopaminergic circuitry is largely spared in this model. This regional specificity aligns with the prominent motor deficits observed in *SNCA* A53T and their improvement following BK40196 treatment. The lack of VMAT1 modulation in both models further supports a selective effect of BK40196 on neuronal (VMAT2-dependent) rather than non-neuronal monoamine storage mechanisms. These new findings reveal a striking contrast between *SNCA* A53T and *LRRK2* G2019S, highlighting fundamentally distinct circuit vulnerability and potential therapeutic responses.

The current data show that BK40196-treated mice had increased D1 receptor levels, suggesting that inhibition of D1 as well as other monoamine transporters results in upregulation of DA levels and enhancement of DA transmission in the mesolimbic and nigrostriatal systems. D1 receptors in the striatum showed no difference in aging mice, also echoing the lack of motor changes in *LRRK2* G2019S. Conversely, D1 staining in the striatum of *SNCA* A53T mice treated with BK40196 was associated with an increase in DA levels and improvement of both motor and behavioral performance. In *LRRK2* G2019S knock-in and BAC transgenic models, reduced striatal DA release and altered cortical DA transmission have been observed prior to cell death, suggesting functional, circuit-specific neurotransmitter dysregulation [22,23]. Similarly, α-synuclein overexpression models, including *SNCA* A53T and A30P, demonstrate pronounced DA deficits in the dorsal striatum, whereas other monoamines remain relatively preserved, indicating selective vulnerability of the nigrostriatal pathway [24,25]. The integration of regional neurotransmitter profiling, behavioral phenotyping, and molecular characterization provides circuit-level resolution of PD pathogenesis. BK40196 alters DA neurotransmitter homeostasis, and represents a highly innovative therapeutic approach with the potential to modify both motor and nonmotor aspects of PD. Other monoamines should also be examined to further dissect the differential contributions of various circuitries in nonmotor symptoms. The findings have strong translational relevance and may guide the development of targeted therapies addressing both motor and nonmotor aspects of PD.

*LRRK2* variants are implicated in both familial and sporadic PD with variable pathology, inconsistent presence of Lewy bodies and marked AD pathology [5]. There is lower incidence of α-synuclein pathology in *LRRK2* carriers [26], but almost 100% of all *LRRK2* PD patients display tau pathology [7,26]. Consistent with human data, our findings indicate that tau hyperphosphorylation is detected in *LRRK2* G2019S mice in an age-dependent manner. Furthermore, PD-risk SNP at the *LRRK2* locus is associated with dysregulated microglial function [27,28]. Notably, experimental and human data indicate that wild-type LRRK2 kinase activity is abnormally increased in idiopathic PD [29] and related models [30], suggesting that inhibition of LRRK2 kinase activity may be neuroprotective. BK40196 provides a strategic option that can target multiple mechanisms via its inhibitory effects on *LRRK2* G2019S together with c-KIT/Abl and FYN, which are associated with neuroinflammation and tau hyperphosphorylation [13]. In addition to pathological mechanisms, *LRRK2* G2019S carriers have unique clinical characteristics, as disease progression is slower compared to sporadic PD [31,32,33], and many individuals carrying the *LRRK2* G2019S mutation do not develop PD despite aging [34]. These data are consistent with the *LRRK2* G2019S model, which does not develop motor and behavioral changes as it ages. BK40196 is superior to other experimental strategies as the heterogeneity of PD necessitates a holistic approach that directly targets inflammation and autophagy as well as *LRRK2* inhibition to address multiple pathologies that are directly or indirectly related to *LRRK2* mutations and/or alpha-synuclein misfolding. Some therapies targeting LRRK2 are under development, but these therapies neglect the multiple pathophysiological mechanisms of *LRRK2*-PD and narrowly focus on inhibition of *LRRK2* mutations only. BK40196 targets validated kinase receptors that harmoniously alleviate inflammation, block tau hyperphosphorylation and promote autophagy [13], while simultaneously inhibiting LRRK2 kinase activity. Engagement of specific and harmonious targets (c-KIT, Abl, DDR1, FYN and LRRK2), among more than 450 kinase targets in the human kinome, is an advantageous approach to significantly reduce peripheral (blood, spleen, etc.) and central inflammation [13,16,35], promote tau de-phosphorylation and improve motor, cognitive and behavioral symptoms in PD and AD models. Compared to idiopathic PD, *LRRK2* mutants have unique characteristics—not only lower motor progression [36,37] but better cognitive performance [38] and slower cognitive decline [31]—and our previous studies in rTg4510 and other transgenic models of AD indicate that BK40196 improves cognitive performance [13].

Clinical studies from our group further support the dissociation between nigrostriatal motor and mesolimbic nonmotor DA pathways in parkinsonism and cognition [14,15]. Treatment of PD and LBD patients with nilotinib or bosutinib significantly increased DA metabolites (HVA, DOPAC) in the cerebrospinal fluid but did not improve motor scores via the Unified Parkinson’s Disease Rating Scale (UPDRS). Instead, these interventions yielded meaningful and significant functional and behavioral improvements, namely via the effects of nilotinib on cognition, consistent with mesolimbic rather than nigrostriatal dopaminergic involvement [14,15]. Together, these findings suggest that mesolimbic dopaminergic signaling represents a key mechanism underlying functional benefits in PD and related synucleinopathies.

Limitations: To control for the effects of multiple targets we used *LRRK2* G2019S versus *SNCA* A53T mice, which displayed significantly different motor behaviors, and both strains were exclusive in expressing their transgene; however, the relative contribution of BK40196 on different targets to the observed in vivo effects remains an area of ongoing investigation.

In conclusion, developing a treatment that can synergistically affect these different pathways is crucial for developing more targeted and efficacious therapeutic interventions. BK40196 is a brain-penetrant molecule with no observed changes in peripheral total or phosphorylated LRRK2, suggesting that it may be a safer treatment for LRRK2 inhibition. BK40196 can be used at very low doses to avoid known peripheral toxicities associated with other LRRK2 inhibitors due to off-target side effects in the lung, kidney and liver. Clinical trials with LRRK2-targeting treatments still need to address several efficacy and safety concerns and trial design challenges. BK40196 can potentially overcome these challenges as a first-in-class molecule that demonstrates abundant brain penetration, central target engagement and efficacy, with the beneficial potential to eliminate side effects.

## Data Availability

The final data, the study protocol and all interpretations will be made available to the scientific and non-scientific community upon request (to C.M.).

## Supplementary Materials

The following supporting information can be downloaded at: https://www.mdpi.com/article/10.3390/biomedicines14040927/s1, Figure S1. BK40196 selectively modulates nigrostriatal dopaminergic markers in *SNCA* A53T mice. Immunohistology staining of *SNCA* A53T mice brain sections and optical density quantification, showing the following: BK40196 treatment increased dopamine D1 receptor expression in the striatum (A,B,G), but not in the nucleus accumbens or SN (H,I) of *SNCA* A53T mice compared to vehicle (DMSO) controls. BK40196 increased dopamine transporter (DAT) expression in the substantia nigra (C,D,L), but not in the striatum or nucleus accumbens (J,K). BK40196 increased vesicular monoamine transporter 2 (VMAT2) expression in the substantia nigra (E,F,O), but not in the striatum or nucleus accumbens (M,N), consistent with improved dopamine vesicular packaging. Expression of VMAT1 remained unchanged across all three regions examined (P,Q,R). Data are presented as the mean ± SD. Table S1. Comparative effects of BK40196 in *LRRK2* G2019S and *SNCA* A53T mouse models across dopaminergic pathways, behavioral outcomes, and experimental timelines.

## Abbreviations

Dementia with Lewy Bodies: DLB; Alzheimer’s disease: AD; Parkinson’s disease: PD; amyloid-beta: Discoidin domain receptor-1: DDR1; hyperphosphorylated tau: p-tau; dopamine: DA; homovanillic acid: HVA; serotonin transporter: SERT; dopamine receptor: D1; dopamine transporter: DAT; norepinephrine transporter: NET; nucleus accumbens: NAcc; substantia nigra: SN.
