# Supplementary material for: Synergistic Effects of Multi-Kinase Inhibition on LRRK2-G2019S and Alpha-Synuclein Pathologies in Models of Parkinson’s Disease
Source: Biomedicines. 2026 Apr 18;14(4):927. doi: 10.3390/biomedicines14040927 (PMC13113530; doi:10.3390/biomedicines14040927)
Supplement: Supplementary file 1 [file biomedicines-14-00927-s001.zip › biomedicines-4177925-Table S1.pdf]

Supplemental Table S1. Comparative effects of BK40196 in LRRK2 G2019S and SNCA A53T mouse models across dopaminergic pathways, behavioral outcomes, and experimental timelines.

Category	LRRK2 G2019S mice	SNCA A53T mice
**Pathological driver**	Mutant LRRK2 kinase hyperactivity	alpha-synuclein overexpression and aggregation
**Age at treatment**	3-4 months<br>8-10 months	8-12months
**Treatment duration**	BK40196 (20 mg/kg, 4 weeks)	BK40196 (20 mg/kg, 4 weeks)
**Primary pathway affected**	Mesolimbic > Nigrostriatal	Nigrostriatal > Mesolimbic
**LRRK2 signaling**	↓ pS1292-LRRK2 (activity marker)	No primary LRRK2-driven change
**alpha-synuclein pathology**	No pathology	↓ syn aggregation
**D1 receptor**	↑ Significant in young and old (NAcc)<br>↑ Significant in young (Striatum)	↑ Significant in Striatum
**DAT expression**	↓ Significant in old (NAcc)	↑ Significant in SN
**VMAT2 expression**	↑ Significant in young (NAcc)<br>↑ Significant in old (SN)	↑ Significant in SN
**VMAT1 expression**	↑ Significant in old (NAcc)<br>↑ Significant in young (SN)	No change
**Dopamine levels**	↑ Significant in young (serum)<br>↓ Significant in old versus young (serum)	↑ Significant in serum
**HVA levels**	↓ Significant in old versus young (serum)<br>↑ Significant in young (brain)	↑ Significant in serum<br>↑ Significant in brain
**Microglia activation**	No change	↑ significant
**TH+ neuron integrity**	No change	↑ significant
**Behavior - motor (rotarod)**	No symptoms detected in young and old	Significant improvement
**Behavior- anxiety (EPM)**	Significant improvement in young	Significant improvement
**Regional specificity**	Mesolimbic dominant	Nigrostriatal dominant
**Mechanistic interpretation**	Likely LRRK2-dependent + multi-kinase effects	Primarily non-LRRK2 (alpha-syn + trafficking pathways)
**Overall response to BK40196**	LRRK2 G2019S specific effects	Multikinase targets<br>C-Kit (autophagy/inflammation)<br>DDR1 (autophagy/inflammation)<br>Fyn (Tau phosphorylation),<br>Abl (autophagy/inflammation)
