# Supplementary figures and images for: Synergistic Effects of Multi-Kinase Inhibition on LRRK2-G2019S and Alpha-Synuclein Pathologies in Models of Parkinson’s Disease
Source: Biomedicines. 2026 Apr 18;14(4):927. doi: 10.3390/biomedicines14040927 (PMC13113530; doi:10.3390/biomedicines14040927)

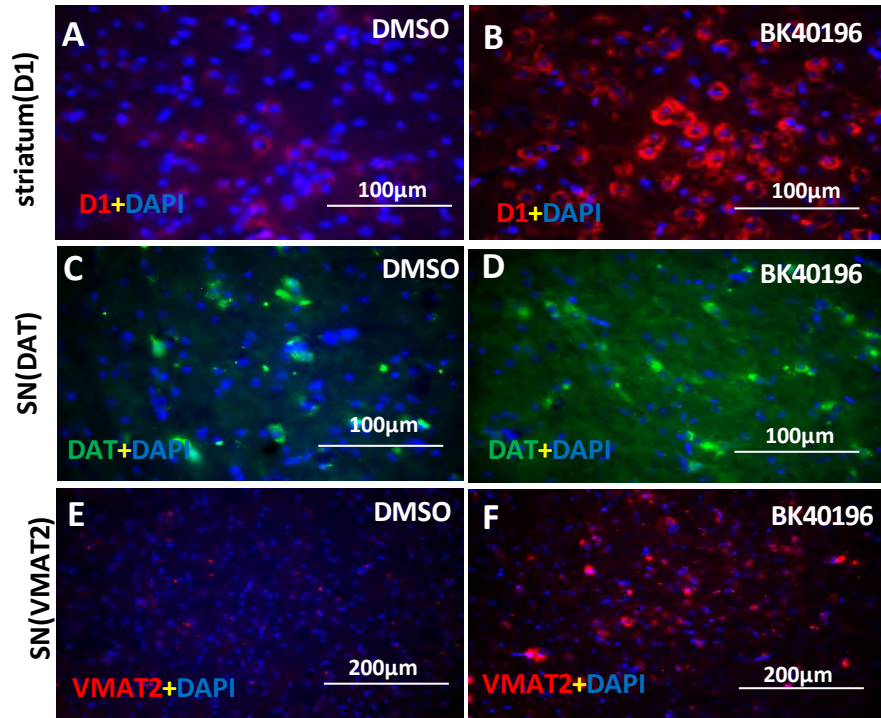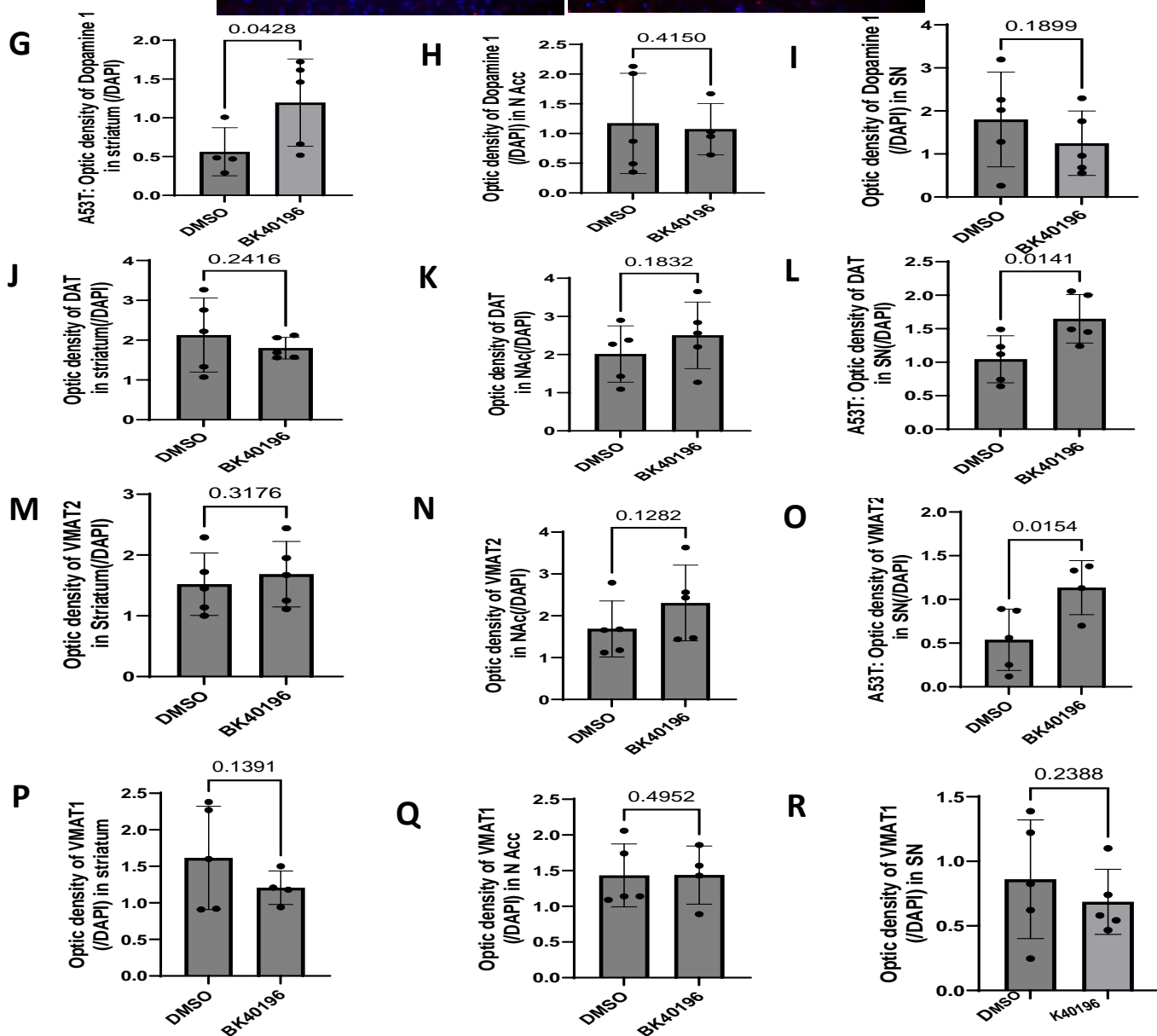

Supplement: Supplementary file 1 [file biomedicines-14-00927-s001.zip › biomedicines-4177925-Figure S1.pdf]
